# Pulsatility of Lenticulostriate Arteries Assessed by 7 Tesla Flow MRI—Measurement, Reproducibility, and Applicability to Aging Effect

**DOI:** 10.3389/fphys.2017.00961

**Published:** 2017-11-24

**Authors:** Roald S. Schnerr, Jacobus F. A. Jansen, Kamil Uludag, Paul A. M. Hofman, Joachim E. Wildberger, Robert J. van Oostenbrugge, Walter H. Backes

**Affiliations:** ^1^Department of Radiology and Nuclear Medicine, Maastricht University Medical Center, Maastricht, Netherlands; ^2^Faculty of Health, Medicine and Life Sciences, School for Mental Health and Neuroscience, Maastricht University, Maastricht, Netherlands; ^3^Department of Cognitive Neuroscience, Faculty of Psychology and Neuroscience, Maastricht University, Maastricht, Netherlands; ^4^Cardiovascular Research Institute Maastricht, Faculty of Health, Medicine and Life Sciences, Maastricht University, Maastricht, Netherlands; ^5^Department of Neurology, Maastricht University Medical Center, Maastricht, Netherlands

**Keywords:** pulsatility, pulsatility index, damping factor, flow quantification, cerebral blood flow, lenticostriate artery, middle cerebral artery, cerebrovascular aging

## Abstract

Characterization of flow properties in cerebral arteries with 1.5 and 3 Tesla MRI is usually limited to large cerebral arteries and difficult to evaluate in the small perforating arteries due to insufficient spatial resolution. In this study, we assessed the feasibility to measure blood flow waveforms in the small lenticulostriate arteries with 7 Tesla velocity-sensitive MRI. The middle cerebral artery was included as reference. Imaging was performed in five young and five old healthy volunteers. Flow was calculated by integrating time-varying velocity values over the vascular cross-section. MRI acquisitions were performed twice in each subject to determine reproducibility. From the flow waveforms, the pulsatility index and damping factor were deduced. Reproducibility values, in terms of the intraclass correlation coefficients, were found to be good to excellent. Measured pulsatility index of the lenticulostriate arteries significantly increased and damping factor significantly decreased with age. In conclusion, we demonstrate that blood flow through the lenticostriate arteries can be precisely measured using 7 Tesla MRI and reveal effects of arterial stiffness due to aging. These findings hold promise to provide relevant insights into the pathologies involving perforating cerebral arteries.

## Introduction

Elevated blood pulsations are thought to contribute to microvascular and tissue damage in the brain. The compliance of the central arteries usually dampens hemodynamic pulsations in an effective way in order to deliver a highly continuous flow to the cerebral microcirculation. However, when arteries stiffen, for instance with aging, the damping becomes less effective and the energy of the pulse waves may be increasingly transmitted to the microvascular system, which may lead to brain tissue damage. In particular, a higher prevalence of subcortical infarcts, atrophy of brain parenchyma, and greater levels of brain amyloid plaques have been found to be associated with increased central artery stiffness and pulsatility (Mitchell et al., 2011; Hughes et al., 2013). The pulsatility of the blood stream increases as the stiffened arterial wall absorbs less pulsatile energy, which leads to less damping of the arterial wave.

Geometry and flow measurements of the intra-cranial arteries are often performed for the larger cerebral arteries including the branches of the circle of Willis. With the emergence of ultra-high field MRI systems, it has become possible to depict the trajectories of the small perforating blood vessels in the brain (Cho et al., 2008). In this study, we imaged the lenticulostriatal arteries (LSAs), which supply the deeper brain structures, including the basal ganglia and parts of the internal capsule. These small perforating arteries are particularly susceptible to damage related to chronic hypertension (Kang et al., 2009). Little is known on the specific effects on the abnormalities of the vessel wall of these arteries. These end-arteries perforate the deep brain tissue and are in direct contact to the brain parenchyma. Amplification of the blood stream pulsations in these arteries might lead to vessel wall alterations and imaging features of cerebral small vessel disease (cSVD) like white matter hyperintensities (Lee et al., 2000; Sierra et al., 2004; Webb et al., 2012; Wardlaw et al., 2013).

A recent ultra-high field study focused on the velocity profile and related pulsatility of the collection of (basal ganglia and semiovale center) perforating arteries in young volunteers, but not individual LSAs and the intracerebral pulse damping effect (Bouvy et al., 2016). Another recent study nicely visualized the velocity and direction of the blood flow averaged over the cardiac cycle in the circle of Willis, major cerebral arteries and LSA in young subjects (Kang et al., 2016). In the current study we focus on the dynamic wave form features (pulsatility and damping) of the flow through the LSA and MCA and on the quality (reproducibility and effect size) of the MRI measurement.

The objectives of this study were threefold: (i) to determine the flow waveform and pulsatility of the LSA and the damping of the pulsations traveling from the middle cerebral artery to the LSAs, (ii) to assess the reproducibility of these quantities, and (iii) to evaluate difference in the damping factor between young and elderly subjects.

## Methods

### Subjects

Ten healthy subjects (five males, 22–84 years) were included in this study. The subjects were equally divided in a younger (age, mean ± SD, 25.0 ± 2.6 years) and older (74.8 ± 5.4 years) group. Neuroradiological evaluation of angiographic scans at 7 Tesla revealed no specific cerebrovascular or other clinically relevant abnormalities of the participants. The study was reported to the medical ethical committee of the institute, which approved the proposal. All participants gave written informed consent to participate in the study.

### Data acquisition

**Figure 1:**
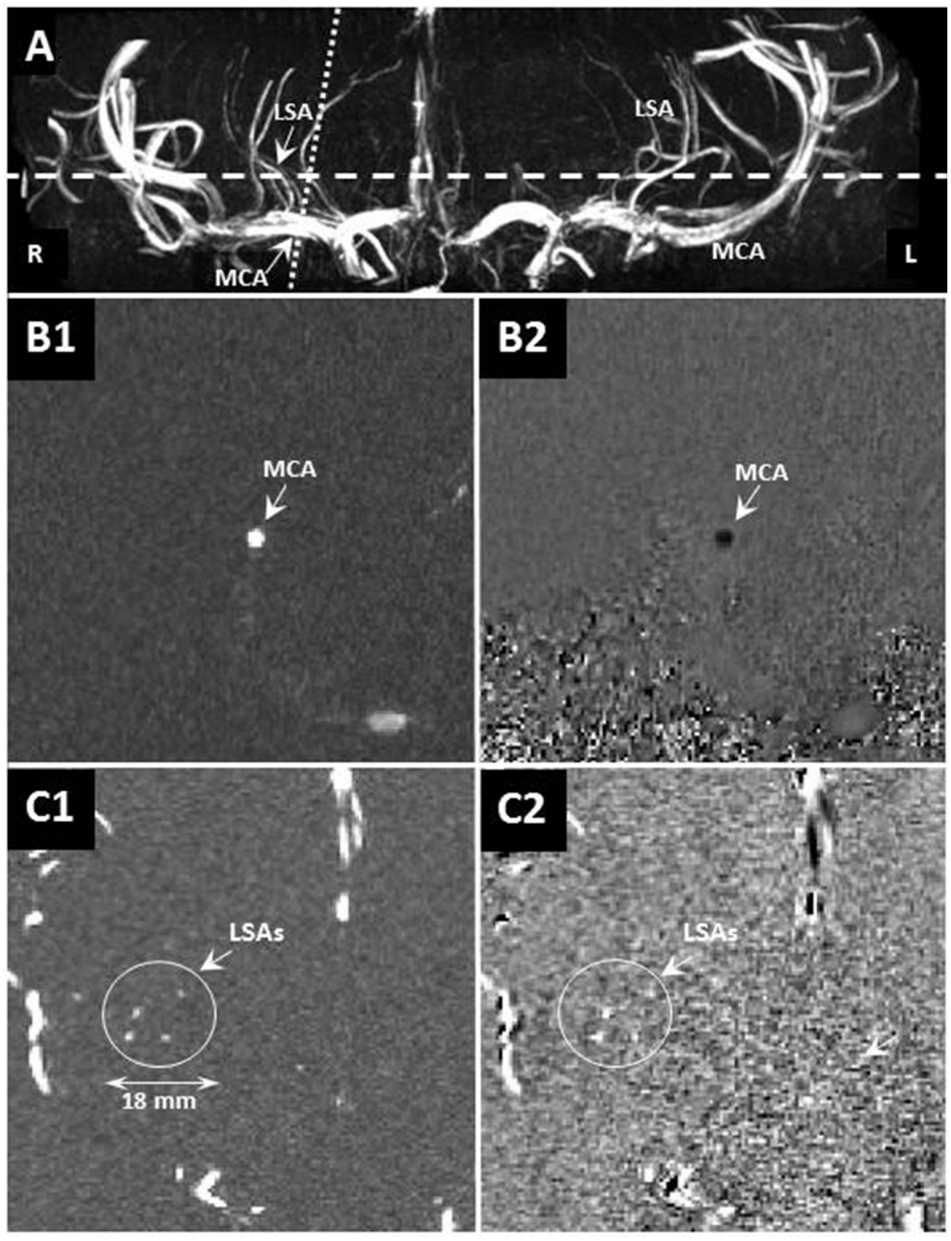
**(A)** Example maximum-intensity-projection of a time-of-flight volume image showing the geometrical planning of the velocity-sensitized slices of the flow waveform measurement in the LSA (dashed line) and MCA (dotted line). **(B)** The velocity-sensitized slices showing the magnitude (left: **B1,C1**) and phase (right: **B2,C2**) of the MCA (top: **B1,B2**) and LSA (bottom: **C1,C2**). The scale is indicated in the bottom left.

A 7 Tesla MRI unit (Magnetom MRI, Siemens Healthcare, Erlangen, Germany) with a 32-channel phased-array head coil (Nova Medical, Wilmington, MA, USA) was used. We applied three-dimensional high spatial resolution time-of-flight (TOF) arteriography to image the intracranial arterial tree. Acquisition parameters for the TOF scan were: repetition time TR = 15 ms, echo time TE = 5.05 ms, flip angle 17°, field-of-view 135 × 180 × 28.1 mm^3^, cubic voxel size 0.22 mm^3^, and scan duration 14 min. On this tree, the middle cerebral arteries and the branching LSAs were identified. The slice for the blood velocity measurements was positioned perpendicular to either the left or right MCA and subsequently to the thickest LSA as close as possible to branching point from the MCA (Figure 1). This slice was 2.0 mm thick, had an in-plane pixel size of 0.47 × 0.47 mm^2^, and was acquired with one velocity-encoding direction along the vessel axis, TR = 41.65 ms, TE = 3.63 ms, and flip angle 17–31° (limited by the specific absorption rate), which was limited by the specific-absorption-rate. The velocity encoding speed (VENC) was set to 100 cm/s for the MCA and 40 cm/s for the LSA to increase the sensitivity for the expected lower flow rate. The measurements were triggered by the cardiac cycle, which was registered with an acoustic sensor positioned in the middle of the thorax at the height of the heart, and fixated with a flexible band around the thorax. On average 21 cardiac phases were acquired for the waveforms (duration 3–4 min). Subsequently, each subject underwent this measurement in the same session a second time including repositioning from the start, as well as planning and acquiring the entire scan once more. The vessels were identified on a second TOF scan and the velocity measurement slices were positioned as described above.

### Image data analysis

Analyses were performed in Matlab (release 2017a 9.2.0; Mathworks, Nattick, MA, USA). For the assessment of the diameters and the waveforms of the MCA and LSA, only the phase images were analyzed. In a few cases, the maximum flow velocity of the MCA peaked slightly above the encoding velocity. We minimized the variance of the velocity as a function of the cardiac cycle to resolve the resulting wrap-around effect. An average velocity map was calculated, and all pixels with a velocity above a threshold (LSA > 3.0 and MCA > 15.0 cm/s) were considered to be part of the vessel. The threshold values were empirically chosen and depended on the noise (velocity) values of the tissue surrounding the vessels, which increased for higher VENC values. Subsequently, the surface area of the vessel was determined by counting the number of pixels of the cross-section at each cardiac phase at half maximum velocity. Assuming a laminar flow profile (i.e., quadratic Hagen-Poiseuille velocity-radius relation), the resulting diameter was then multiplied by a factor √2 to correct for the outer shell region (i.e., radial position > vessel radius/√2). The waveforms of the LSAs visible in each measurement were averaged, and to reduce the noise of the waveforms, these were smoothed using a 5-point Bartlett filter. The flow waveforms were calculated by integrating the velocity values over the vessel cross-sections. For the diameter and flow, the mean, minimum, and maximum values over the cardiac cycle were denoted. For the LSAs, the number of arteries analyzed was also reported, where the analysis for the MCA was restricted to one. The three flow values were combined to the (blood flow) pulsatility index (PI) by dividing the difference between the maximum and minimum value to the mean flow value (Gosling and King, 1974). The damping factor (DF) was calculated as the ratio of the PI values of the MCA and LSA (Gosling and King, 1974).

### Statistical analysis

Diameter and flow values were reported using mean values and standard deviations. The mean diameter and flow values of the younger and older group were compared by means of an independent Student *t*-test. Statistical significance was inferred when *p* < 0.05.

The test-retest reproducibility of the measurement technique was evaluated with (i) the coefficient of repeatability (CoR), which is the average variation over the two measurements (relative to the mean value in %) times 1.96 and the (ii) intra-class correlation coefficient (ICC), which expresses the variance between subjects relative to the sum of between-measurement and between-subject variance. The ICC over the two repeated measurements was calculated as ICC = (BMS–WMS)/(BMS+WMS), where BMS and WMS are the between-subjects and within-subjects mean squares, respectively (Shrout and Fleiss, 1979). The following classification was used: ICC < 0.40 poor, 0.40 ≤ ICC < 0.60 fair, and 0.60 ≤ ICC < 0.75 good, and 0.75 ≤ ICC ≤ 1.00 as excellent (Cicchetti, 1994). The ICC was calculated over the entire study group, as it removes any differences in the mean values between the two groups by scaling of the measured values. In contrast, the CoR was calculated separately over the younger and older group to deal with possible differences in mean values. Furthermore, Bland-Altman graphs were constructed, in which the differences between the two measurements were plotted as a function of the mean, and inspected for possible trends (Bland and Altman, 1986).

## Results

### Caliber

**Figure 2:**
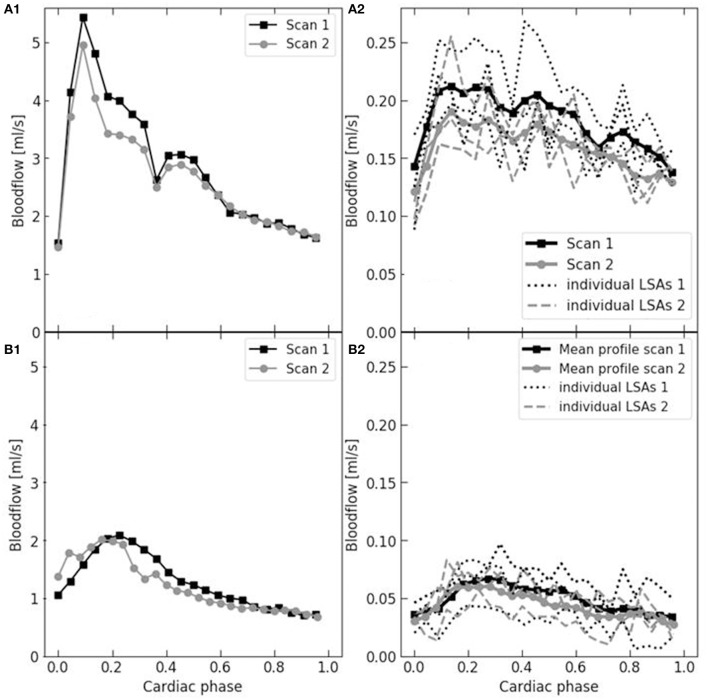
Example flow waveforms in the MCA (left: **A1,B1**) and LSA (right: **A2,B2**) and of a young (upper: **A1,A2**) and older (bottom: **B1,B2**) subject. The two subsequent reproducibility measurements are depicted (black squares and gray circles, respectively). The waveform of the MCA was derived from one vessel and, for the LSA, it was averaged over multiple vessels (three in these cases). Note that the MCA waveform of the older subject is lower and less sharp.

Figure 2 shows examples of the flow waveforms of the MCA and LSA for each age group. In the older subjects, the waveform of the MCA is less sharp, more broadened, compared to the younger subjects. Also, it can be noted from Figure 2 that the strength of the pulsation is quite lower in the LSAs compared to the MCAs.

The diameters of the MCA and LSA were 3.3 ± 1.3 mm (mean ± SD) and 1.7 ± 1.2 mm, respectively, averaged over all 10 subjects. The diameters varied a factor of 1.3 and 1.6 over the cardiac cycle for the LSA and MCA, respectively (Table 1). There were no significant differences in vessel diameters between younger and older subjects. Also note the variation over the cardiac cycle, which indicate that these vessels are not completely stiff in older subjects.

**Table 1:** Arterial diameters (mm) over the waveform and reproducibility of their mean values.

**Artery**	**Measure**	**Young subjects**		**Old subjects**		**ICC**
		**Diameter (mm)**	**CoR [mm (%)]**	**Diameter (mm)**	**CoR [mm (%)]**	
MCA	Mean	3.4 ± 1.5	1.3 (38)	3.2 ± 0.6	1.5 (45)	0.73(0.22–0.92)
	Min	2.4 ± 1.4		2.3 ± 1.3		
	Max	4.0 ± 1.6		3.8 ± 1.1		
LSA	Mean	1.7 ± 0.9	0.82 (48)	1.7 ± 1.4	1.3 (76)	0.76(0.29–0.93)
	Min	1.4 ± 0.7		1.5 ± 1.3		
	Max	1.9 ± 1.0		1.9 ± 1.4		

*Arterial diameters did not significantly differ between young and old subjects. Diameter notation: mean ± standard deviation*.

*CoR, Coefficient of Repeatability; notation, absolute value (percentage of mean value); ICC, Interclass Correlation Coefficient; notation, value (95% confidence interval)*.

### Flow and pulsatility

**Figure 3:**
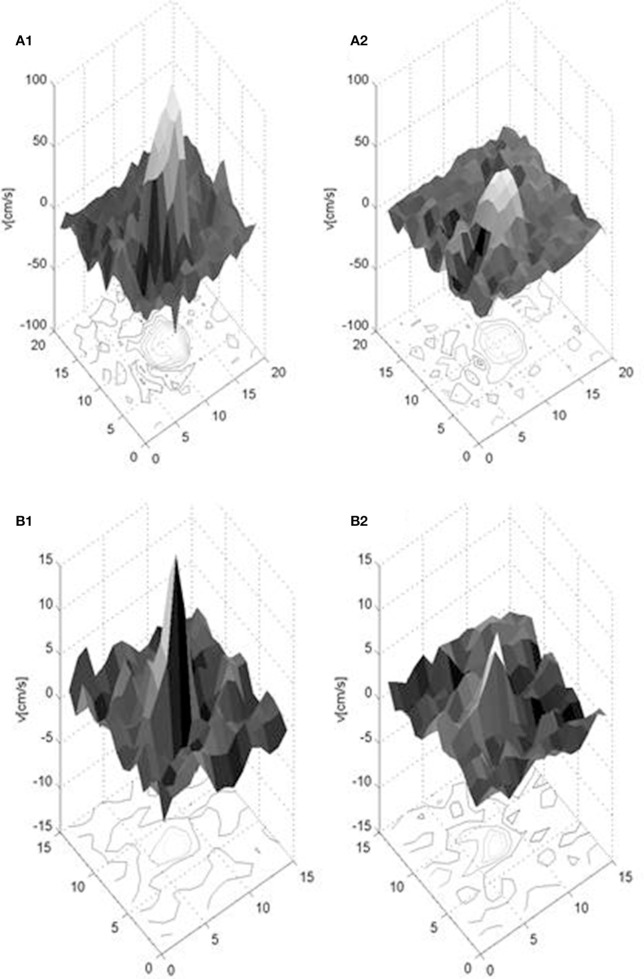
Exemplary spatial velocity profiles in a young healthy subject for the MCA (upper: **A1,A2**) and LSA (lower: **B1,B2**) for two cardiac phases, when the velocity has its maximum (left: **A1, B1**) and minimum (right: **A2,B2**) value. Note the different velocity scales along the vertical axes. The units of the two horizontal axes are in pixels and the depicted surface mesh shows the pixel size of the measurement.

The derived measures of the flow waveforms, flow, pulsatility index, and damping factor, are listed in Table 2. Mean peak (minimum–maximum) during cardiac cycle blood velocities were 61.4 (42.8–92.4) cm/s for the MCA and 8.2 (6.2–10.1) cm/s for the LSA in the younger subjects, and 39.2 (26.0–56.4) cm/s and 4.5 (2.9–6.1) cm/s respectively in the older subjects. The number of LSAs, in which the waveform could be obtained, were between 3 and 7 in the younger subjects and between 3 and 4 in the older subjects. Figure 3 shows examples of spatial velocity profiles across the vascular cross-section.

**Table 2:** Flow, pulsatility index and damping factor and reproducibility of their mean values.

**Artery**	**Measure**	**Young subjects**		**Old subjects**		**ICC**
		**Flow (mL/s)**	**CoR [mL/s (%)]**	**Flow (mL/s)**	**CoR [mL/s (%)]**	
**FLOW WAVEFORM**						
MCA	Mean flow	**2.56 ± 0.63**	**0.83 (32)**	**1.48 ± 0.30 *p* = 0.011**	0.15 (10)	0.84 (0.47–0.96)
	Min flow	**1.59 ± 0.49**		**0.96 ± 0.28 *p* = 0.036**		
	Max flow	**4.19 ± 0.67**		**2.22 ± 0.48 *p* = 0.001**		
LSA	Mean flow	0.10 ± 0.04	0.02 (18)	0.05 ± 0.03 *p* = 0.073	0.03 (54)	0.91 (0.70–0.82)
	Min flow	0.08 ± 0.03		0.04 ± 0.03 *p* = 0.077		
	Max flow	0.12 ± 0.05		0.07 ± 0.03 *p* = 0.087		
**DERIVED QUANTITIES**						
MCA	PI	1.12 ±0.39	0.47 (42)	0.88 ± 0.22 *p* = 0.27	0.31 (35)	0.68 (0.13–0.91)
LSA	PI	**0.46 ± 0.05**	0.17 (37)	**0.69 ± 0.22 *p* = 0.05**	0.24 (35)	0.74 (0.25–0.93)
MCA 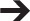 LSA	DF	**2.40 ± 0.76**	0.98 (41)	**1.31 ± 0.30 *p* = 0.017**	0.61 (46)	0.73 (0.22–0.92)

*All MCA waveform flow measures (mean, min, max) were significantly lower for the older subjects compared to the younger subjects (p < 0.04). For the LSA, there was a comparable trend (p < 0.01). The pulsatility index (PI) was significantly higher for the older subjects for the LSA (p = 0.05), but not for the MCA (p = 0.27). The damping factor (DF) was significantly lower for the older subjects (p = 0.017). Statistical tests (p-values) were performed by a Student's t-test*.

*Flow notation, mean ± standard deviation; statistical significance is indicated by BOLD type setting. CoR, Coefficient of Repeatability, notation, absolute value (percentage of mean value); ICC, Interclass Correlation Coefficient, notation, value (95% confidence interval)*.

### Reproducibility

The reproducibility values of the diameter assessment are listed in Table 1. The ICC was at the border between good and excellent (0.73 for MCA and 0.76 for LSA). The CoRs were 0.97 and 1.15 mm for the MCA and LSA, respectively, thus quite comparable in magnitude considering the pixel size (0.47 × 0.47 mm^2^) used.

In Table 2, the reproducibility values of the flow measurements are specified. The ICC of the mean flow measurements were excellent for both the MCA and LSA, and the CoR values (young/old: 0.83/0.15 mL/s for MCA and 0.02/0.03 for LSA) were smaller than the differences between the younger and older subjects (mean flow difference between young and old: 1.08 mL/s for MCA and 0.05 mL/s for LSA). For the PI, good ICC values for both arteries were obtained, with CoR values comparable to the difference between the two age groups. Also for the DF, good ICC values were obtained, with a CoR quite smaller than the group difference.

**Figure 4:**
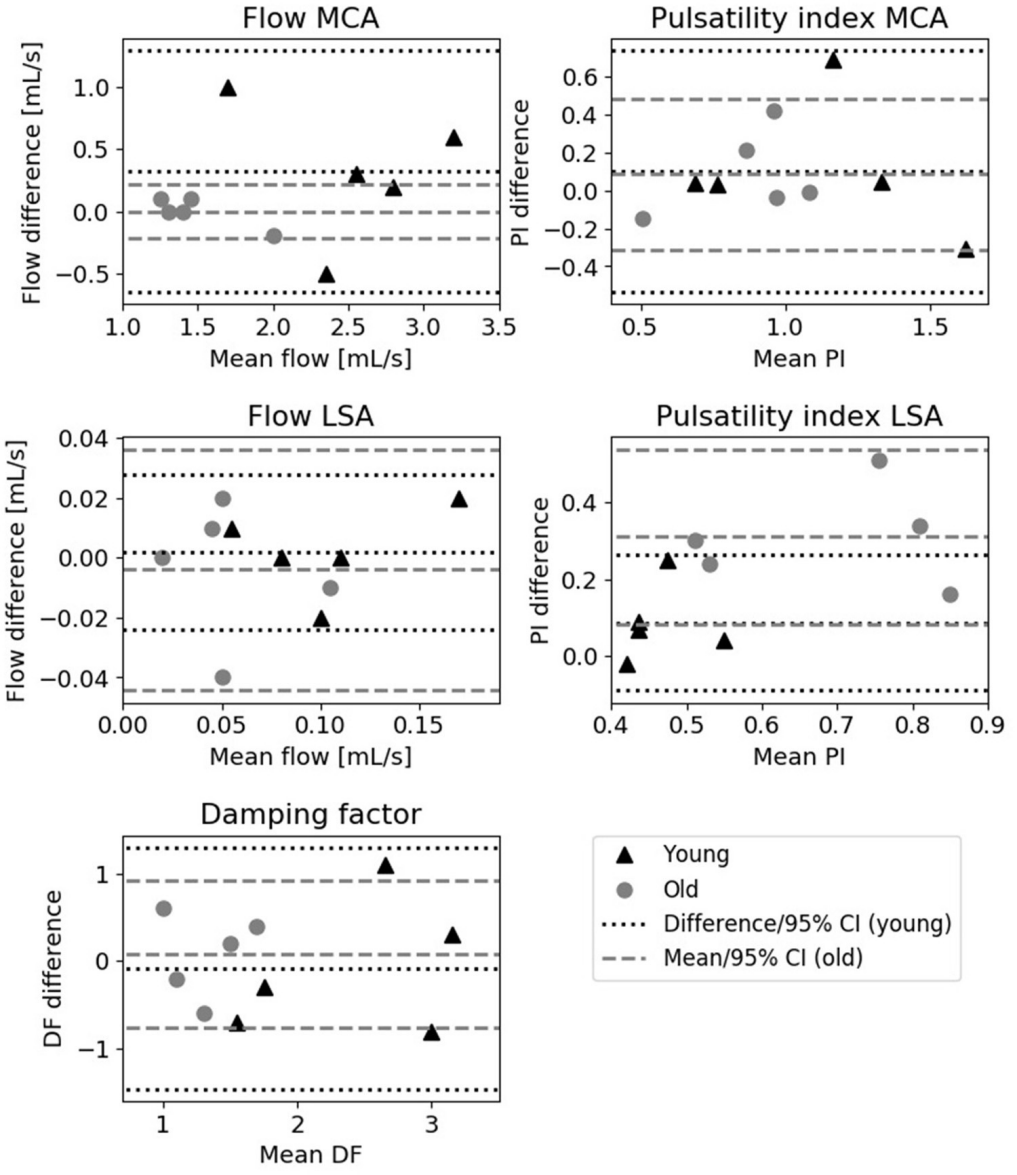
Bland-Altman plots showing the difference between the two measurements vs. the mean. Depicted are the mean flow and pulsatility index of the MCA (**upper row**), mean blood flow and pulsatility index of the LSA (**middle row**) and damping factor measurements (**bottom row**) for young (triangles) and old (circles) subjects. The lines indicate the mean and 95% confidence interval of the measurements.

Figure 4 shows the Bland-Altman plots of the various flow derived quantities. All 95% confidence intervals were situated around zero and no systematic differences between the two measurements were observed for the flow derived quantities as a function of the magnitude.

### Aging effects

From Table 2 also effects of aging can be derived. The mean MCA flow was approximately 42% lower in the older subjects compared to the younger subjects (*p* = 0.011). Also the time-resolved minimum and maximum flow values through the MCA were significantly lower for the older subject group. For the LSA, also a trend lower flow values (though not statistically significant) were found in the older subjects, see Table 2.

The PI of the older subjects in the LSA, but not the MCA, was significantly higher than for the younger subjects (0.69 vs. 0.46, *p* = 0.05). For the PI, the MCA showed quite strong intersubject variations. For the older subjects, the DF of the arterial flow pulse from the MCA to the LSA was significantly lower than for the younger subjects (1.31 vs. 2.40, respectively, *p* = 0.015).

## Discussion

In this study, we set out to measure the calibers and blood flow of the lenticulostriate arteries and their reproducibility in the human brain using 7 Tesla MRI. Furthermore, we evaluated whether differences between young and old healthy subjects can be observed in terms of blood flow and pulsatility in the MCA and LSA and damping of the pulse along this trajectory. Key observations were that (i) the flow measurements and the derived quantities can be obtained in a highly reproducible way and that (ii) the pulsatility in the LSA and intracerebral damping of the blood pulse wave from the MCA to the LSA was significantly lower in the older subjects.

### Calibers

The MCA diameter values obtained by the current technique (3.4 ± 1.5 mm) correspond very well with values reported in the literature. For instance, using black-blood MRI, Serrador et al. (2000) found 2.5 ± 0.6 mm and Verbree et al. (2016) measured 3.1 ± 1.2 mm. Also the results of Stock et al. (2000), who used phase-contrast MRI and found a diameter of 2.8 ± 1.2 mm, agree to our findings. Although there are quite a number of articles on the imaging of the trajectories of the LSAs, no reports exists to our knowledge on the caliber assessment. It should be noted that the small diameter of 1.7 mm of the LSA is at the border of what is technically possible using flow measurements considering the CoR (0.82 mm) and pixel size (0.47 × 0.47 mm^2^).

### Flow and pulsations

Our MCA mean flow values on the younger subjects (2.52 ± 0.56 mL/s) fit well to the results of Zarrinkoob et al. (2015), who found 2.68 ± 0.52 mL/s using MR flow measurements in a group of 25 year old subjects. However, their flow values in the elderly (mean age 71 year) were on average 32 % higher (2.18 ± 0.38 mL/s) than ours (1.48 ± 0.30 mL/s). Such deviations may arise between study groups, for instance, due to differences in cardiac output, cardiovascular health and/or cerebral tissue volume. In a study with carotid obstructions, Stock et al. (2000) found values of 1.6 ± 0.5 mL/s in subjects over 60 years and 2.1 ± 0.5 mL/s in subjects of 30 years or younger. Our mean flow results fall well within the variation of results found in the literature.

We are not aware of any flow measurements in the LSAs. Though Bouvy et al. (2016) measured at 7 Tesla field strength the velocity profiles in healthy young subjects in the collection of arteries in the basal ganglia and the MCA, no flow values were reported. In that study, the mean velocity value (4.6 cm/s) in the basal ganglia arteries (LSAs and its branches) of young subjects (mean age 27 years) was quite lower than mean LSA velocity (8.2 cm/s) of the younger subjects in our study. This lower velocity might be due the selection of more and especially smaller (basal ganglia) arteries and the (possibly non-perpendicular) angulation of these arteries with respect to the imaging slice. The mean LSA velocity values of Kang et al. (mean 9.6 cm/s, range 7–12 cm/s; mean age 33 years), fit better to our results, although these were (cardiac-cycle average) maximum values over the arterial trajectories.

### Reproducibility

With the current study, we obtained highly satisfying test-retest reproducibility values for the arterial diameters and blood flow, pulsatility, and damping factor value assessments. The CoR value of the diameter measurement is in the order of 1 mm, which is approximately equal to two pixels, and indicates that it resides at the border of what is achievable in terms of spatial resolution with current flow measurements. For the flow measurements, similarly good to excellent results were obtained as for the velocity measurements in a previous reproducibility study (Bouvy et al., 2016). The precision of the current technique is relevant for follow-up studies, in which cerebrovascular pulsatility effects of aging or treatment are studied, for instance in patients with cerebrovascular disease or other disorders including hypertension, diabetes, and (prodromal stages of) dementia. This reproducibility study is helpful for this, as it provides for the first time the smallest noticeable physiological differences in terms of blood flow, pulsatility, and damping factor of healthy young and older subjects.

### Vascular aging

In the current feasibility study, we show that the effect of arterial aging can be well demonstrated with 7T flow waveform measurements. We found significant differences for the MCA flow, pulsatility index of the LSA and damping factor over intracerebral trajectory from the MCA to the LSAs. Pulsatility was increased in the older subjects, which was partly due to a somewhat lower mean LSA flow and partly due to stronger dynamic flow variations, i.e., larger difference between maximum and minimum flow in the cardiac cycle. The increased pulsatility index in the older subjects can be explained by the hardening of the arterial wall and an increased resistance of the cerebral (micro)vasculature. Also the mean blood flow decreases with age, due to an increased cerebrovascular resistance, and therefore adds to the increase of the pulsatility index. The effect of elevated pulsatility index arises also due to a decreased wave damping by the intracerebral arterial tree, and complies with the general notion that the elastic component of the vessel walls are lost during (natural) aging. For the older subjects, this means that the kinetic energy of blood ejected by the heart is less dampened by the Windkessel effect of the vessels and more energy is deposited to the cerebral microcirculation and brain tissue. In this context, it is relevant to note that we measure the waveforms in the LSAs, which are perforating arteries that are in close contact to the brain tissue. It has furthermore been shown that increased stiffness of the aorta may cause features of cerebral small-vessel-disease (cSVD), such white matter hyperintensities and subcortical infarcts (Mitchell et al., 2011). Future studies are required in cSVD populations to demonstrate the link between arterial LSA stiffness and subcortical, cortical and white matter tissue damage (Fisse et al., 2016). Previously, Zarrinkoob et al. (2016) also demonstrated an increased (MRI-derived) pulsatility index in various large cerebral arteries with aging, including the MCA (0.89 vs. 0.72, in 71 vs. 22 year old healthy subjects), which agrees well with our results.

### Methodological considerations

The current study represents a methodological feasibility study to quantify anatomical and functional properties of the LSAs, promising that arterial aging effects can already be detected with a small number of subjects. We want to emphasize that the focus of this work lies on exploration of what is technically possible. Primary interest was not in quantification of the magnitude of biological differences between subgroups, which in our view can better be left for future confirmatory studies. Such future studies should base sample size calculation on the smallest clinically relevant difference that is worthwhile to detect, which can be different from the technical uncertainties we reported on (Pocock, 1983). Larger populations are also required to discern differences between various age groups, sex, other demographic characteristics and cardiovascular risk factors. With the inclusion of young and old subjects we obtained a range of flow related values, and not just values in a relatively small range as for young subjects. The results of the reproducibility assessment showed that the test-retest error (precision) of the measurement is smaller than the observed aging effect.

We furthermore assumed a laminar velocity profile to determine the hemodynamic vessel diameter, which is valid for flow in normal blood vessels (beyond bifurcations, stenosis, etc.), to which the low velocity values close to the noise level near the vessel walls are not very relevant. For non-laminar flow conditions (turbulent or plug flow), i.e., situations we do not expect, this approach is expected to be less accurate. The technique of waveform registration at 7 Tesla MRI can be further optimized by increasing the number of cardiac phases, increasing the spatial resolution, optimizing the image quality, and increasing the total acquisition duration in cooperative subjects.

## Conclusions

We showed cardiac-cycle-resolved caliber and flow measurements in the small perforating arteries in the brains of young and old subjects using 7 Tesla MRI, and showed the feasibility, to measure the blood flow waveforms not only through the MCA but also of the smaller LSAs in a reproducible way. Our results demonstrate aging changes in the pulsatility index and damping factor in these arteries, which appear to reflect the arterial hardening properties of the aging cerebrovasculature. This development at ultra-high-field MRI paves the way to more specifically investigate the effects of hardening of the perforating arteries in contrast to central or peripheral vascular effects of aging in disease conditions, such as diabetes and hypertension and also other vascular complications.

## Ethics statement

This study was carried out in accordance with the recommendations of the Medical Ethical Trial Centre with written informed consent from all subjects, in accordance with the Declaration of Helsinki. The protocol was approved by the Medical Ethical Trial Centre of the Maastricht University Medical Centre.

## Author contributions

RS, PH, KU, RvO, and WB: the conception or design of the work; RS and WB: the acquisition, analysis and the interpretation of data for the work, and drafting the work; RS, JJ, KU, PH, JW, RvO, and WB: final approval of the version to be published; RS, RvO, and WB: agreement to be accountable for all aspects of the work; RS: the acquisition of data for the work; PH: clinical evaluation; rest: the design of the work; revising the work; final approval of the version to be published; agreement to be accountable for all aspects of the work; WB, JJ, JW, and RvO: adjustments due to review.

### Conflict of interest statement

The authors declare that the research was conducted in the absence of any commercial or financial relationships that could be construed as a potential conflict of interest.
